# Safety and effectiveness of an innosvative SS-suction device to control moisture in dental procedures

**DOI:** 10.1016/j.heliyon.2023.e18129

**Published:** 2023-07-08

**Authors:** Sukanya Tianviwat, Kan Pokawattana, Songchai Thitasomakul

**Affiliations:** a Evidence-Based Dentistry for Oral Health Care and Promotion Phase II Research Unit, Faculty of Dentistry, Prince of Songkla University, Hatyai, Songkhla, 90110, Thailand; b Department of Preventive Dentistry, Faculty of Dentistry, Prince of Songkla University, Hatyai, Songkhla, 90110, Thailand; c Residency Training Program in Dental Public Health, Faculty of Dentistry, Prince of Songkla University, Songkhla, 90110, Thailand

**Keywords:** Sealant, Suction, Device, Moisture control, Isolation

## Abstract

Control of moisture is critical for retention of sealants, which can reduce the incidence of caries in high-risk groups. Objectives: We investigated the safety and efficacy of the novel SS-suction device in the laboratory and a small clinical trial in children aged 6 to 8-years-old. Methods: First, a laboratory test of 52 SS-suction devices was conducted to determine the pressure generated by the chin plate to maintain suction at different intervals and to determine the pressure resistance of the spring to maintain suction in a child's mouth. Second, 12 healthy children with sound lower molars participated in a clinical trial of the use of SS-suction during sealant application. Results: The laboratory test showed that when pressed to the maximum distance of 25 mm, the chin plate produced a pressure of 247.5 ± 116.6 mmHg. At this pressure, the SS-suction could be used safely for up to 120 min without damaging the soft tissues. In the clinical study, the dentists inserted and removed the SS-suction in 7.80 ± 2.48 s. The range by which the chin plate was pressed down varied between 0 and 13 mm. The highest pressure on the skin was 120 mmHg (at 13 mm) and the device effectively maintained suction and effectively removed water and saliva. The time required to apply sealant was 7.01 ± 2.26 min. Conclusions: The SS-suction is a safe, effective device for two-handed application of sealants in children, with no negative side effects. Clinical significance: We demonstrate the unique SS-suction device can quickly drain water and saliva to help dentists treat young patients using a two-handed approach; this device provides good control of moisture during sealant application. The device prevents the tongue and cheeks from interfering with the operation area, reduces tension, and does not require a dental assistant.

## 1 Introduction

Moisture control is critical to the efficacy and success of various dental procedures, especially resin sealants [1,2]. Properly applied sealants provide effective protection against caries; however, the surface to be treated must be clean and dry [1,3]. Loss of sealant due to poor moisture control leads to a higher incidence of caries in the long-term [4]. To improve the retention of resin-based sealants, adequate moisture control can be achieved by employing effective moisture control devices and having a skilled dental assistant use a high-volume evacuator to prevent moisture contamination, or through the use of glass ionomer sealants [5,6]. Therefore, appropriate isolation devices that effectively suction away water and saliva are critical to the efficiency of treatment by improving sealant retention to prevent caries [7]. The American Academy of Pediatric Dentistry (AAPD) recommends that dental assistants administer suction, i.e., a four-handed technique, during the application of pit and fissure sealants if dental facilities have enough dental professionals [8]. If dry isolation is problematic on the area to be sealed and no dental assistant is available to assist with suction, the AAPD recommends the use of a more hydrophilic substance, glass ionomer. However, a resin-based sealant may be more desirable if the tooth can be isolated to ensure long-term retention [9]. Overall, effective moisture control is desirable to increase the preventative effects of resin-based dental sealants.

However, most healthcare institutions, particularly government facilities in developing countries, frequently lack sufficient numbers of dental assistants to satisfy demand [10,11]. In developing countries, dental services without dental assistants are not uncommon. The two-handed technique is more commonly used in outreach services than the four-handed technique with a dental assistant. In Thailand, dental care is not available in remote areas, such as health promotion hospitals in sub-districts, where most facilities do not have dental assistants. A dental assistant is not always present during treatment, even in developed countries. Moreover, four-handed sealant delivery is more expensive than two-handed sealant delivery [12,13]. Thus, an effective isolation device that can deliver comparable moisture management outcomes is required for one-operator techniques.

When a dental assistant is not available, dedicated suction equipment should be set up to enable dental personnel to treat patients successfully and reduce functional stress during specific dental treatments that require substantial moisture management, such as application of resin sealants. To address this problem, this study was initiated to evaluate an innovative, high-volume saliva tube called SS-suction, which can rapidly evacuate water and saliva while providing good visibility. The suction tips of this device also prevent the tongue and cheek from interfering with the operating area, thus making dental procedures more comfortable.

The SS suction device was recently designed as a self-retaining suction tip with the goal of allowing dental personnel to work without the assistance of a dental assistant, compared to traditional suction tips that require a dental assistant. The device was originally designed and manufactured to perform moisture control, retract the cheek, and prevent the tongue from entering the operation area in children 6–8 years of age, especially in conjunction with serious moisture control procedures such as pit and fissure sealing of the lower first permanent molar.

**Fig. 1:**
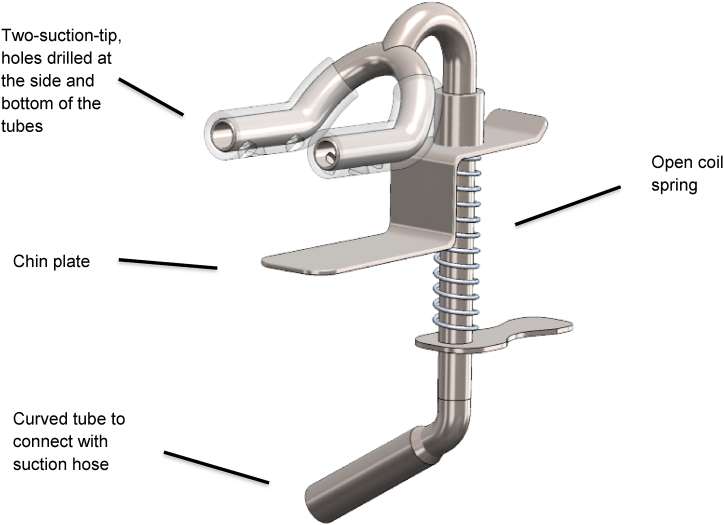
Features and composition of the SS-suction device.

The SS-suction consists of three parts (Fig. 1): the first part is the tips of the saliva ejector, which are designed to fit the contour of the mandible. The tips of the two saliva tubes are placed parallel to the alignment of the teeth. The tips of the saliva tubes are covered with silicone rubber to reduce the pressure exerted by the tips of the saliva ejectors on the soft tissues of the mouth. The bottom of the saliva tube has holes through which saliva can be aspirated from the floor and vestibule of the mouth. These holes promote faster saliva flow when rinsing teeth or drilling teeth with an air turbine handpiece during dental procedures. When the SS-suction is connected to a high-volume evacuator with a wind speed of 100 cfm, the water flow rate is 200 mL per minute. The second component of the device is a metal chin plate that is attached under the child's chin and can be stabilized via spring pressure to hold the SS-suction in position with the mandible. The height of the chin plate can be adjusted according to the height and size of the jaw. The spring force holds the chin plate in position to keep the suction device in place without damaging the skin on the chin. The position of the suction tube can be changed by rotating and adjusting the chin plate. The third component is a curved extension tube that connects to the saliva suction tube on the dental chair. These components are designed as one piece and cannot be separated. The suction consists of a 304-grade stainless steel tube that can be easily disinfected and sterilized with a steam sterilizer.

Suction devices must be tested to ensure their safety and effectiveness before they can be used on a large scale [[14], [15], [16]]. The phases of medical device testing include: 1. initial exploration of the risks and opportunities associated with medical device development; 2. design and development. 3. assessment of patient safety during the use of the device; 4. preparation for product launch and final testing; and 5. product launch and post-launch support. It is necessary to study the safety of SS-suction both *in vitro* and *in vivo* before it is used in larger groups. Therefore, a laboratory safety test and a small clinical trial of SS-suction were conducted. The purpose of this study was to evaluate the spring compression pressure of the SS-suction in the laboratory and to investigate the efficacy and identify any adverse effects related to the use of the SS-suction during sealant application in a small group of children aged 6–8 years.

## 2 Materials and methods

### 2.1 Sample and design

This experimental study was divided into two parts. The first trial was a laboratory study to determine the spring pressure of 52 SS-suction tubes, and the second trial was a clinical study of the use of SS-suction in 12 healthy children aged 6–8 years with healthy deep pits and fissures of the lower first molars. The children were selected from two primary schools in a low-fluoride suburban area with average socioeconomic status in Thailand (average per capita income of 4730 USD). The five girls and seven boys had good oral hygiene; the school's oral health services in this area are provided by Hatyai Reginal Hospital, Thailand [17]. The researcher explained the procedure of the experiment to the children and their parents. Any unclear details related to the study procedure were clarified until the participants and their parents or guardians understood everything. The participants and their parents/guardians signed a document agreeing to participate in the study. This study was approved by the Ethics Committee of the Faculty of Dentistry, Prince of Songkla University, EC6206-016. The “SS-suction” and “2-way SS-suction” were granted a petty patent on January 26, 2022, by the Department of Intellectual Property of Thailand under number 2003000542.

### 2.2 Determination of the spring pressure of the SS-suction in the laboratory

**Fig. 2:**
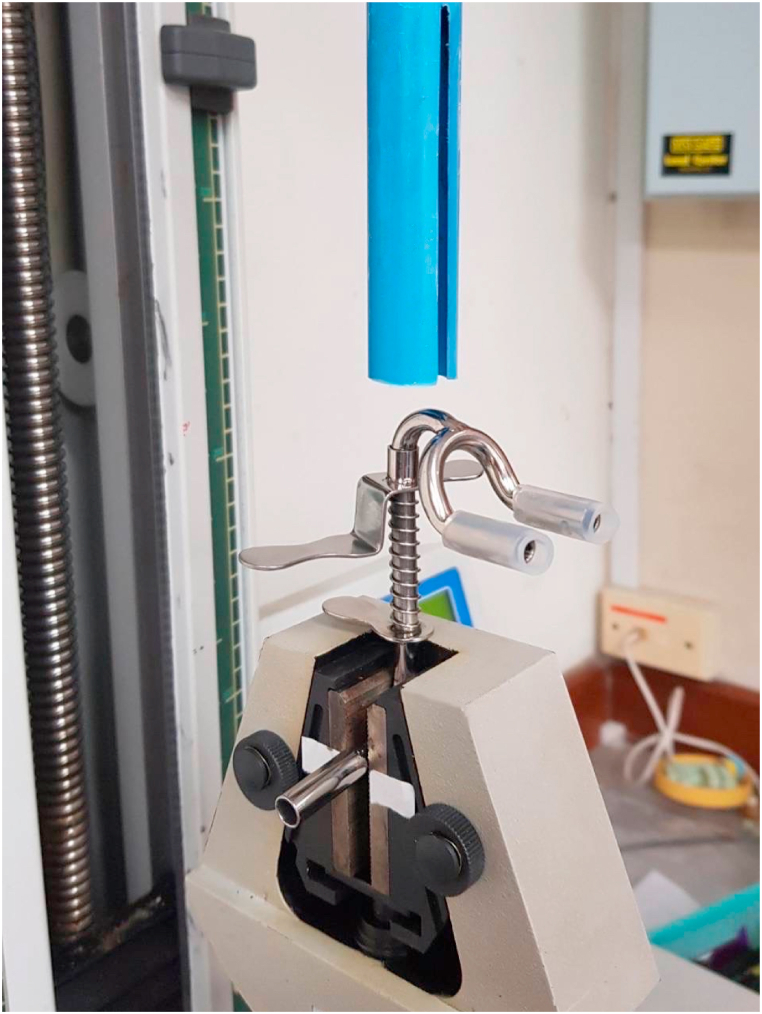
Determination of the spring force of the SS-suction using a universal testing machine.

**Fig. 3:**
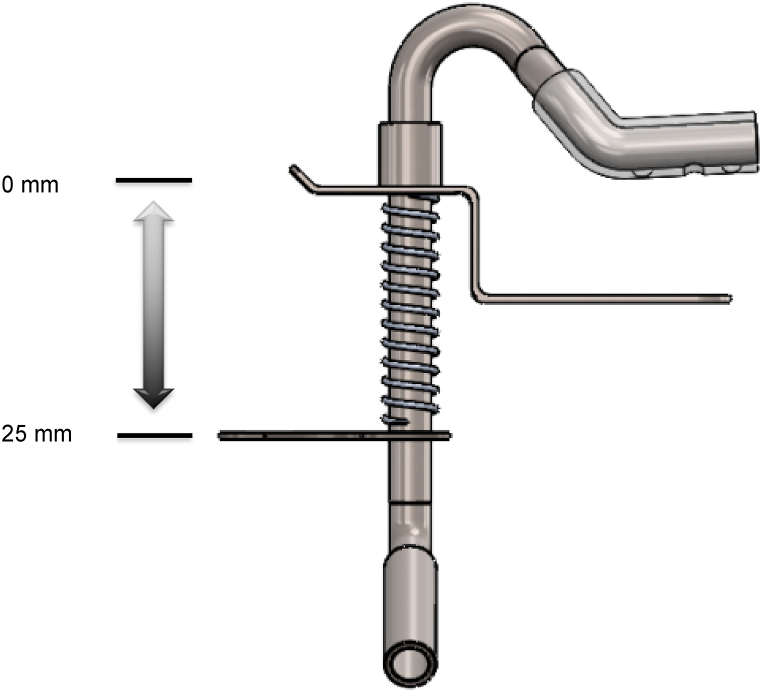
Schematic showing the chin plate of the SS-suction can be pressed down by 25 mm.

**Fig. 4:**
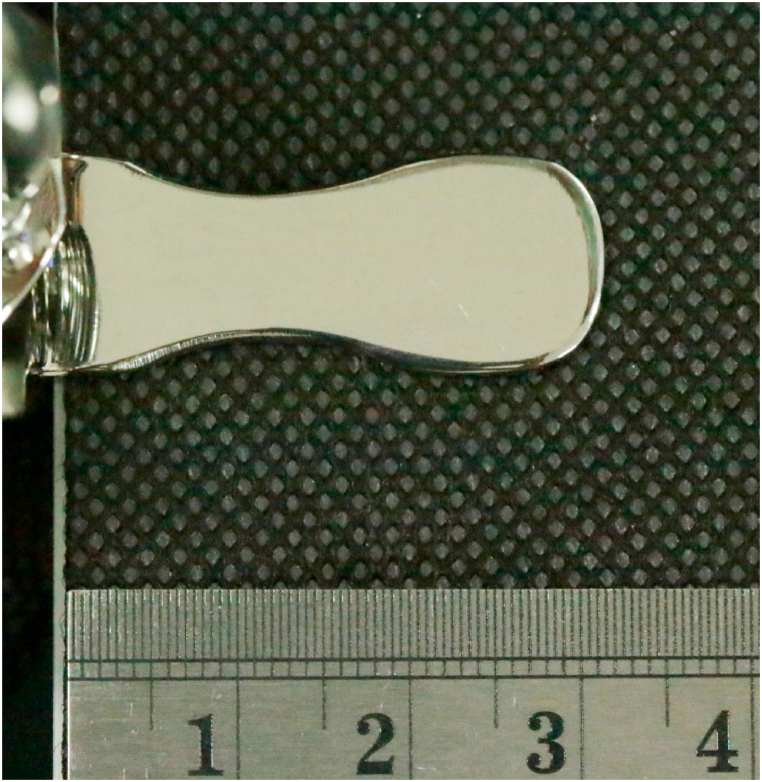
Image of analysis of the area of the chin plate of a SS-device (scale in centimeters).

**Fig. 5:**
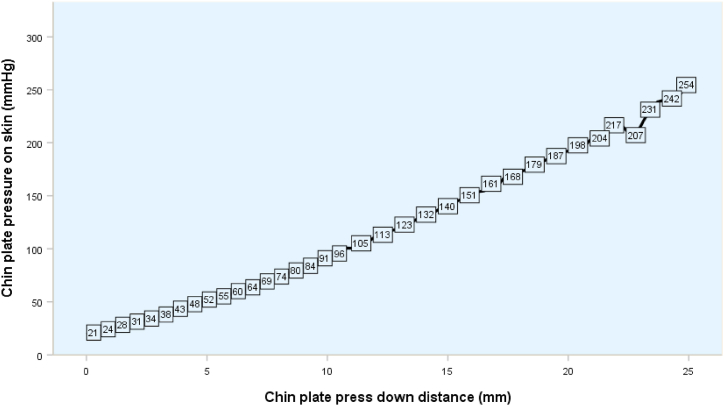
Pressure exerted on the skin depending on the chin plate press-down distance of the SS-suction.

In the first trial, one author (KP) used a LRX-Plus universal testing machine (AMETEK-Lloyd Instrument Ltd., England) to measure the spring retention force of the SS-suction. The SS-suction was attached to a jack stand (Fig. 2). The universal testing machine was used to gradually increase the pressure on the chin plate of the suction. The open coil spring was pushed down from 0 to 25 mm to simulate the action of dental personnel pushing down on the chin plate, taking the suction into the child's mouth, and releasing the chin plate to hold the suction to the child's chin. The resistance force of the spring against the testing machine was recorded from 0 mm when the spring was not depressed up to 25 mm when the spring was fully depressed (Fig. 3). The instrument transferred and recorded the resistance force of the spring and the distance the spring was compressed and plotted a graph showing the relationship between the resistance force and the distance the spring was compressed in Nexygen® software. Each SS-suction was tested three times, and the average resistance force was used for data analysis. However, as the effect of pressure on the chin plate on the child's chin varies depending on the size of the chin plate, we determined the size of the chin plate by photographing the chin plate alongside a scale ruler (Fig. 4), imported the image into Zen Blue® software, and calculated the area of the chin plate in square millimeters. Then, the spring force was divided by the size of the chin plate to obtain the pressure in units of Newton/mm^2^. The pressure unit was then converted to mmHg, as 1 N/mm^2^ is equal to 7500.63 mmHg. Finally, the pressure data were used to plot a graph showing the relationship between the distance the chin plate was pressed down and the pressure exerted by the chin plate of the SS-suction device on the skin (Fig. 5).

### 2.3 Analysis of the safe duration of use of the SS-suction

**Fig. 6:**
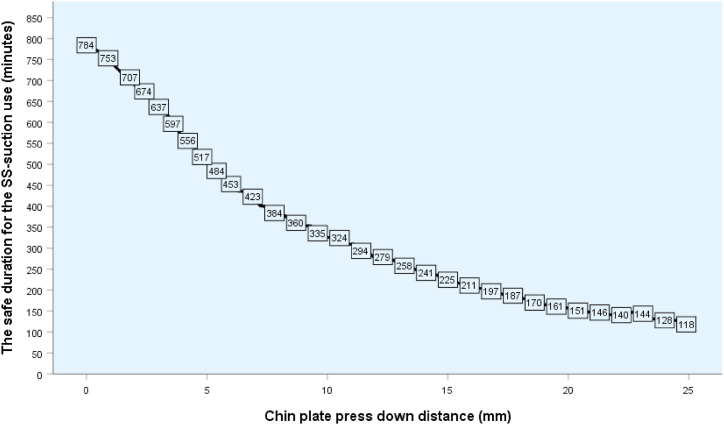
Duration of safe use of the SS-suction according to the chin plate press-down distance.

After calculating the average force generated by the spring of the chin plate, we determined the safe duration of application of the SS-suction device without damage to soft tissue, based on the threshold pressure values previously described by Lowthian [18]. Lowthian stated that 0.5-h continuous compression at a pressure of 480 mmHg was safe and 1-h compression at 350 mmHg was safe. The chin plate of the SS-suction device could safely compress soft tissue for longer at lower pressure levels, e.g., at pressure levels of 270, 230, 170, 120, 80, 70, 60, 50, 40, 30, 20 mmHg, the safe application time would be 1.2, 1.5, 2.0, 3.0, 4.0, 5.0, 6.0, 8.0, 10.0, 11.5, and 13.0 h, respectively. Using these data, a graph was created showing the relationship between the chin plate distance and the safe duration of use of the SS-suction (Fig. 6).

### 2.4 Clinical study of the SS-suction in a small group of children

**Fig. 7:**
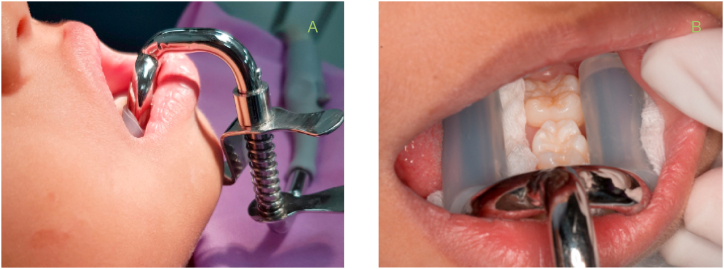
Trial of the SS-suction device in children. A, spring pressure held the SS-suction in position with the mandible without the assistance of a dental assistant. B, During the sealing process, the two ends of the SS-suction effectively retracted the tongue and cheek, and saliva and water were quickly evacuated, keeping the area around the molars dry.

As the SS-suction device passed the first study and the spring pressure met the required standards without a risk of damaging the oral soft tissue or the skin under the chin, two experienced dentists applied sealants to 12 children aged 6–8 years (Fig. 7) using the SS-suction device. Pit and fissure sealing was the main procedure in this study. As per the requirement of the ethics committee, it was mandatory for a dental assistant to attend these procedures, to help in case of emergency and prevent negative consequences. The duration of time for which the SS-suction was inserted for sealing was recorded. In addition, the chin plate press-down distance of the SS-suction was measured. During the sealing process, the ability of SS-suction tips to protect the tongue and cheek and the degree of obstruction of the operational field caused by the suction body were also recorded. The patients were monitored for signs of gagging or breathing difficulties during application of the device. After application of the device, we assessed for the presence of soreness, pressure points, bruising, pain, and discomfort in the chin and neck.

### 2.5 Data analysis

All data were checked for completeness and accuracy. All variables were imported into a computer, including the time required to insert and remove the SS-suction device from the children, the incidence of sores, bruising, pain, and discomfort in the chin and neck area, gagging, and respiratory disturbances during the use of SS-suction. The data were analyzed using IBM SPSS Statistics (version 29.0) to determine the duration of safe use and the occurrence of abnormal events during or after the use of the SS-suction device.

## 3 Results

### 3.1 Pressure exerted by the chin plate

Spring pressure increased when the open coil spring was pressed downward in a laboratory test of 52 SS-suction devices (Fig. 5). When no pressure was placed on the chin plate, the initial pressure was 0 mmHg. When the spring was pressed, the pressure gradually increased. The chin plate can be pressed down by a maximum of 25 mm, resulting in a pressure of 247.5 ± 116.6 mmHg.

### 3.2 Recommended safe duration of use for the SS-suction device

The average surface area of the chin plate was measured at 318 ± 25 mm^2^. Fig. 6 shows an analysis of the pressure exerted on the jaw by the chin plate when the spring is depressed during use of the device for mouth suction. We found that for small children or children with a small jaw, the compression distance of the spring is also small, and the device only places light pressure on their chin. However, the chin plate needs to be pressed down further for larger children with larger jaws, and thus a higher pressure is exerted on the chin. When the chin plate is pressed down less than 10 mm, the pressure on the chin is moderate and the suction device can be used safely for up to 320 min, while stronger pressure needs to be placed on the chin plate for larger children and the suction device can be used safely for only 150 min. The pressure values determined in these experiments ranged from 0 to 254 mmHg, which demonstrates that the device can be used safely when then chin plate is depressed by 0–25 mm. Even when the maximum pressure is exerted by depressing the chin plate by 25 mm, the maximum time the suction can be retained in the mouth without damaging the skin is 120 min (Fig. 6).

### 3.3 The SS-suction device provides effective moisture control

The trial applications of the SS-suction device showed that the operators could insert and remove the SS-suction device from the children's mouths within 4–10 s. The average duration was 7.80 ± 2.48 s, including the time required for the operator to connect the suction tube to the device, push down the chin plate, insert the SS-suction tip into the mouth, and adjust the position until the device was ready for use. The removal time begins when the operator pushes down on the chin plate and removes the SS-suction from the child's mouth. The trials also confirmed that the pressure exerted by the spring onto the chin plate holds the suction to the jaw without the need for additional help from a dental assistant. The suction device could be easily inserted or removed by pressing and adjusting the chin plate. The SS-suction could be removed quickly without injuring the child and no emergencies occurred during this clinical study. The entire process of applying dental sealant while using the SS-suction device took only 3–12 min, with an average of 7.01 ± 2.26 min. The good patient cooperation when using SS-suction during the sealing process shortened the working time. During sealing, the operators found that the operation field was clear because the two ends of the suction effectively retracted the tongue and cheek. In addition, saliva and water were quickly evacuated, keeping the area around the molars dry and clean and allowing the operator to perform the sealing procedure without interruptions due to changing cotton rolls and gauze pads.

In this clinical study of the SS-suction, the chin plate was pressed down 0–13 mm depending on the size of the jaw of the children. In some cases, the chin plate did not need to be depressed (0 mm) and the suction device could remain securely in the oral cavity without the pressure spring effect. In cases where the chin plate compression distance was 13 mm, the pressure exerted on the skin by the chin plate was 120 mmHg. At this pressure level, the device could be used for up to 4 h without damaging the children's soft tissue (Fig. 6). Moreover, no adverse effects such as injuries, bruising, or redness in the mouth, chin, or face of the children occurred during this clinical trial. The chin plate of the suction does not extend to or place pressure on the neck area, and thus does not affect breathing. Inserting the SS-suction device into the children's mouths did not cause gagging. Overall, the operators could work more easily and quickly with the dental sealant, even during rinsing, because the SS-suction efficiently evacuated water and saliva.

## 4 Discussion

This study demonstrates that the SS-suction is an innovative, self-retaining suction device that effectively controls moisture and is safe for use on children. The SS-suction can also prevent the tongue and cheek from entering the operation area and effectively reduces saliva contamination during dental procedures. We investigated the efficiency and safety of this newly invented suction tube that has not yet been used in dental practice. We assessed the ease of insertion and removal and the safe duration of use without damaging the soft tissue. This self-retaining suction tube can be attached to the mandible of children aged 6–8 years by pressing a spring to secure a chin plate; therefore, the device was tested in a small cohort to demonstrate the SS-suction does not disrupt breathing. The results showed that insertion and removal of the suction tube is simple, taking less than 10 s (average of 7.80 ± 2.48 s) for one operator. This confirms that the use of the SS-suction is convenient, simple, and considered safe.

The chin plate that holds the SS-suction can gently move up and down and rotate relative to the suction tube, which makes it easy to adjust the position of suction in the mouth. Due to the spring pressure on the chin plate attached to the mandible, the SS-suction is designed to be maintained in the mouth itself. The test was conducted to ensure that the suction pressure would not damage either the intra-oral or extra-oral tissues. The chin plate pressure was assessed in relation to the duration of dental treatment to determine the appropriate duration of safe use. We found that the use of SS-suction was safe for all pediatric treatment procedures. The chin plate pressure was moderate and could be used for more than 2 h, which is sufficient for a variety of dental procedures, and there was no incidence of tissue damage during the study. The clinical duration for sealing teeth is usually 12.5 min per tooth [19]. In the current study, the operators needed only 7 min to complete the sealing of one tooth, which is lower than the general average. This could be due to the fact that the SS-suction not only removes moisture, but also protects the tongue and cheek from interfering with the dental procedure. The protection of the child's cheek, the good visibility of the operation field, and the fact that there is no need to change cotton rolls or gauze during treatment all contribute to faster work. A previous study reported that children are willing to undergo re-sealing because of their good experience during the application of dental sealants [20]. Sealants appear to be effectively prevent caries and are a cost-saving treatment in children at high risk of caries [[21], [22], [23]]. This is consistent with previous studies in which Isolite was used as a saliva-repellent device to seal pits and fissures in molars in an average of 10 min [24]. However, the clinical treatment time spent on dental work depends on the oral characteristics and cooperation of the child.

To protect children from damage or injury during use, the suction tips of the SS-suction have silicone tubes on both sides. The soft silicone tubes reduce the pressure exerted on the oral soft tissues. Therefore, it was not surprising that no injuries or red bruising were observed after use of the SS-suction in the current study. However, we recommend that gauze or cotton rolls should be placed under both ends of the suction tube to better control moisture. However, the operator does not need to change the gauze or cotton rolls during the procedure, which is more convenient and requires less treatment time than conventional approaches. The children's good cooperation with the treatment is beneficial and leads to higher treatment efficiency. In this study, there was no occurrence of skin lesions on the chin after the application of the suction device, which could be due to the low pressure exerted by the round chin plate, which does not have sharp edges. None of the children expressed discomfort as a result of the use of suction during the application of dental sealant. The length and width of the chin plate are designed to hold the suction device in place without obstructing the children's throats, so that their breathing is not affected and gagging did not occur when the SS-suction was used. Indeed, some of the children in this study choked easily when water was sprayed to rinse their teeth, but not when the SS-suction was used.

Thus, the SS-suction provides advantages over traditional methods of tooth isolation, such as the use of a cotton roll, gauze pad, or rubber dam. These advantages include the ease of insertion and removal. The device is secured in the mouth using a chin plate that is held in place using light spring pressure. The design of the suction tubes, which curve along the dentition and buccal vestibule, prevent the tongue and cheek from interfering with the process of sealing the lower molars. The SS-suction drains water and saliva effectively, making it suitable for application of dental sealants without the need to change gauze or cotton rolls during treatment. In terms of disadvantages, one child had a deep floor of the mouth, which caused the suction device to tilt to the lingual side. The dentist adjusted the position of the chin plate to match the child's oral anatomy. However, as the child's jaw was very small, the suction device did not hold well in the child's mouth and the operator had to hold the suction while working. In addition, because the suction is a foreign object in the mouth, some children pushed the device with their tongues. In order to work efficiently, the operator sometimes had to hold the suction device in place. No negative side effects were observed in this study.

The SS-suction is intended to be one-size-fits-all device that can be used on both the right and left sides of the mandible. This study demonstrates the device is suitable for most children aged between 6 and 8 years and is therefore very practical, as different sizes of device are not required. In addition, the SS-suction is made of 304-grade stainless steel and is therefore reusable and can be easily cleaned with soap, disinfected, and sterilized in an autoclave, which saves resources. For children with jaws that are very small or very large, the operator can rotate or reposition the suction to fit the anatomy of the child's mouth by tilting or aligning the suction at a slight angle toward the tongue. To adapt the suction to the floor of mouth and buccal vestibule, the operator may need to place gauze and cotton rolls under the suction tips for some children or adjust the plane of the suction tube on both sides so that the suction tubes are angled toward the floor of mouth. These simple adjustments can improve the performance of the saliva tube. It may also be necessary to move the suction tube slightly forward or backward to effectively draw water and saliva. Placing cotton rolls or gauze pads at an appropriate height under the saliva tube allows the suction tube to work more effectively.

## 5 Conclusions

The SS-suction is a simple and safe device for use in children, with no harm or adverse reactions observed. The suction device can effectively control moisture and there is no need to change cotton rolls or gauze pads during treatment. The SS-suction also prevents obstruction of the operative field by the tongue and cheek and reduces saliva contamination. Therefore, this device can be proposed for widespread production and clinical use, as well as further evaluation of user satisfaction. Further experiments should be conducted with other types of dental treatments that require strict moisture control, especially in situations where there is a shortage of dental assistants.

## Author contribution statement

Sukanya Tianviwat; Kan Pokawattana and Songchai Thitasomakul: Conceived and designed the experiments; Performed the experiments; Analyzed and interpreted the data; Contributed reagents, materials, analysis tools or data; Wrote the paper.

## Data availability statement

Data will be made available on request.

## Declaration of competing interest

The authors declare that they have no known competing financial interests or personal relationships that could have appeared to influence the work reported in this paper.
